# Cytisinicline vs. Varenicline in Tobacco Addiction: A Literature Review Focused on Emotional Regulation, Psychological Symptoms, and Mental Health

**DOI:** 10.3390/healthcare13151783

**Published:** 2025-07-23

**Authors:** Óscar Fraile-Martínez, Cielo García-Montero, Miguel A. Ortega, Andrea Varaona, Luis Gutiérrez-Rojas, Melchor Álvarez-Mon, Miguel Ángel Álvarez-Mon

**Affiliations:** 1 Department of Medicine and Medical Specialities, Faculty of Medicine and Health Sciences, Network Biomedical Research Center for Liver and Digestive Diseases (CIBEREHD), University of Alcalá, 28801 Alcala de Henares, Spain; oscar.fraile@uah.es (Ó.F.-M.); cielo.garcia@uah.es (C.G.-M.); varaonaandrea@gmail.com (A.V.); mademons@gmail.com (M.Á.-M.); maalvarezdemon@icloud.com (M.Á.Á.-M.); 2 Ramón y Cajal Institute of Sanitary Research (IRYCIS), 28034 Madrid, Spain; 3 Psychiatry and Neurosciences Research Group (CTS-549), University of Granada, 18012 Granada, Spain; gutierrezrojasl@hotmail.com; 4 Immune System Diseases-Rheumatology and Internal Medicine Service, University Hospital Prince of Asturias, Networking Research Center on for Liver and Digestive Diseases (CIBEREHD), 28806 Alcala de Henares, Spain; 5 Department of Psychiatry and Mental Health, Hospital Universitario Infanta Leonor, 28031 Madrid, Spain; 6 CIBERSAM-ISCIII (Biomedical Research Networking Centre in Mental Health), 28029 Madrid, Spain

**Keywords:** smoking cessation, cytisinicline (cytisine), varenicline, nicotinic acetylcholine receptors (nAChRs), emotional regulation, neuropsychiatric safety, tobacco dependence

## Abstract

Tobacco use disorder remains a leading cause of preventable mortality, with nicotine playing a central role in the development and maintenance of dependence, mainly through its action on α4β2 nicotinic acetylcholine receptors (nAChRs). Smoking cessation treatments must address both physiological withdrawal and the affective disturbances (such as anxiety, irritability, and mood lability) which often facilitate relapses. This review compares two pharmacotherapies used in smoking cessation, varenicline and cytisinicline (cytisine), with particular focus on their impact on emotional regulation, psychological symptoms, and neuropsychiatric safety. Varenicline, a high-affinity partial agonist at α4β2 nAChRs, has demonstrated superior efficacy in maintaining abstinence and is well-supported by robust clinical data, including in psychiatric populations. However, its use may be limited by adverse effects such as nausea and sleep disorders. Cytisinicline, a structurally similar but less potent partial agonist, has recently gained renewed interest due to its lower cost, favorable tolerability profile, and comparable effectiveness in the general population. Although less extensively studied in patients with serious mental illness, preliminary data suggest cytisinicline may offer a better side effect profile, particularly regarding sleep disturbances and emotional reactivity. Both agents appear to ameliorate withdrawal-related affective symptoms without significantly increasing psychiatric risk. Ultimately, pharmacotherapy choice should be guided by individual clinical features, mental health status, treatment tolerability, and resource availability. Further research is needed to establish cytisinicline’s efficacy and safety across diverse clinical contexts, particularly among individuals with severe psychiatric comorbidities.

## 1. Introduction

Tobacco use remains one of the leading preventable causes of morbidity and mortality worldwide, accounting for over 8 million deaths annually. This figure includes approximately 1.3 million non-smokers who die due to exposure to second-hand smoke [1].

Nicotine, one of the most active and addictive components of tobacco smoke, binds strongly to neuronal nicotinic acetylcholine receptors (nAChRs), particularly the α4β2 subtype. These receptors are densely expressed in the brain’s reward circuitry, where nicotine’s actions contribute to the development and persistence of tobacco dependence and addiction [2].

However, tobacco use disorder is not purely a neurochemical addiction. It also functions as a maladaptive form of emotional regulation [3,4]. Many smokers report using nicotine to manage negative emotions, relieve stress, and reduce symptoms of anxiety and depression [5,6,7]. Withdrawal from nicotine is often accompanied by affective symptoms such as irritability, dysphoria, insomnia, and heightened anxiety, which are major contributors to relapse, especially during the early stages of abstinence [8].

From this perspective, smoking becomes a dual-process phenomenon: driven by neurobiological reinforcement and sustained by affective vulnerability. Emotional dysregulation, in turn, may both trigger smoking and undermine cessation efforts [9].

This understanding highlights the importance of pharmacological agents that not only target physical dependence but also modulate emotional processes. Nicotinic partial agonists, including varenicline and cytisinicline, selectively modulate α4β2* nAChRs, attenuating withdrawal symptoms while blunting nicotine’s reinforcing effects [10,11]. These agents not only reduce cravings but may also help stabilize mood during abstinence, potentially through dopaminergic modulation [12,13].

Despite similar mechanisms, their pharmacokinetic profiles and tolerability differ, potentially influencing emotional outcomes, adherence, and psychiatric safety in distinct ways.

Among them, varenicline—approved by the FDA in 2006—has long been considered the most effective first-line monotherapy. Despite its efficacy, it is often underprescribed, and adherence rates remain suboptimal [12,14,15]. Concerns regarding tolerability may also impact both adherence and treatment outcomes. Although varenicline is generally safe, some studies have reported adverse effects, including rare but serious neuropsychiatric symptoms [7,12,16,17].

Cytisinicline (also known as cytisine), an older but recently re-evaluated compound, appears comparably effective and may present a more favorable tolerability profile in some studies [18]. While it has been used in Eastern Europe for decades, high-quality randomized controlled trials have only recently been conducted in North America and Western Europe [19]. The neuropsychological and emotional impacts of these medications, however, remain underexplored, particularly in populations with mental illness or heightened affective reactivity.

Indeed, smoking rates and nicotine dependence are disproportionately high in individuals with psychiatric disorders (1.7 to 3.3 times the rate of the general population), who also face more significant barriers to cessation due to emotional dysregulation and vulnerability to relapse [20,21,22]. Thus, understanding how varenicline and cytisinicline differ in their impact on mood, anxiety, irritability, and neuropsychiatric safety is critical to optimizing treatment strategies for this high-risk group.

This review critically examines and compares varenicline and cytisinicline, focusing on their effects on emotional regulation, mood symptoms, neuropsychiatric safety, and treatment adherence. By integrating findings from pharmacological, neurobiological, and clinical perspectives, we aim to clarify their respective roles in addressing both the physical and affective dimensions of tobacco dependence.

Special attention is paid to high-risk clinical subgroups—including those with psychiatric comorbidities, low treatment adherence, or emotional dysregulation—to support more targeted and personalized cessation strategies.

## 2. Methodology

This narrative review was designed to provide a theory-informed synthesis of current evidence comparing cytisinicline and varenicline for smoking cessation, with a particular emphasis on emotional regulation, affective symptoms, and psychiatric safety. Unlike systematic reviews that quantitatively aggregate effect sizes, a narrative approach was chosen to allow a more flexible and conceptually guided exploration of emerging themes—especially those concerning mental health subpopulations and neuropsychological mechanisms. The review was organized into three main thematic sections: (1) an overview of pharmacological characteristics and mechanisms of action of both agents along with their general efficacy in smoking cessation; (2) implications for emotional regulation and affective symptoms; and (3) neuropsychiatric safety and specific applications in populations with mental disorders or emotional vulnerability. This thematic structure allowed for the integration of diverse study types and perspectives within a unifying framework focused on the intersection between pharmacotherapy and mental health.

The literature search was conducted using databases such as PubMed, Scopus, and Web of Science, focusing on publications from the last two decades (since the approval of varenicline). Keywords included “cytisinicline”, “cytisine” “varenicline”, “tobacco cessation”, “mental health”, “emotional regulation”, and “psychological symptoms”. No strict methodological design filters were applied, as the objective was to incorporate a wide range of evidence types, including clinical trials, observational studies, previous reviews, and conceptually relevant neuropsychological analyses. Inclusion was based on (1) peer-reviewed empirical studies, meta-analyses, or reviews in English or Spanish; (2) studies involving human participants or preclinical models providing valuable mechanistic insights into the role of cytisinicline or varenicline; and (3) relevance to at least one of the following: therapeutic efficacy, psychological or psychiatric effects, or pharmacological comparisons between cytisinicline and varenicline. Studies were excluded if they were unpublished, conference abstracts, or non-peer-reviewed sources. The synthesis was narrative, integrating findings thematically to explore similarities, differences, and gaps in the existing literature.

## 3. Cytisinicline and Varenicline: An Overview

Both cytisinicline and varenicline act as partial agonists at nicotinic acetylcholine receptors. Varenicline binds selectively and with high affinity to the α4β2* nAChR subtype, producing only ~40–60% of nicotine’s dopamine-releasing effect [23]. Although varenicline also exhibits weak affinity for 5-HT_3_ receptors, its clinical efficacy is primarily driven by α4β2 activity [24]. This partial agonism reduces cravings while simultaneously blocking nicotine’s full agonist effects.

Cytisinicline, which is structurally similar, also acts as a partial agonist at α4β2* receptors but exhibits significantly lower binding affinity [23]. Thus, both medications moderately stimulate α4β2 receptors to alleviate withdrawal symptoms and competitively inhibit nicotine, thereby reducing its reinforcing effects.

Boths drugs are taken orally in form of pills or tablets. Varenicline is well absorbed (food-independent), with peak plasma levels in ~3–4 h and a long elimination half-life (~24 h) [14]. It undergoes minimal metabolism with 92% excreted unchanged in the urine. Steady-state conditions are reached in ~4 days of dosing.

By contrast, cytisinicline has a much shorter half-life (≈4.8 h) [25]. In humans, a single 1.5 mg dose gives a C_max of only ~15.5 ng/mL at ~1 h, and ~64% of an oral dose is excreted unchanged in urine within 24 h [26]. Cytisinicline has moderate oral bioavailability (~42%) and limited brain penetration (brain levels ~30% of plasma). It is cleared rapidly via renal and biliary routes.

Varenicline is typically prescribed for 12 to 24 weeks. The standard adult regimen begins one week prior to the designated quit date: 0.5 mg daily on days 1–3, 0.5 mg twice daily on days 4–7, and then 1.0 mg twice daily from day 8 onward (continued for 12 weeks) [27]. An additional 12-week extension is often recommended to sustain abstinence. Behavioral counseling is given concurrently.

Cytisinicline is prescribed over a shorter period, typically around 25 days. A commonly used regimen involves taking 1.5 mg tablets six times daily (about every 2 h) for the first 3–4 days, followed by a gradual taper to two doses per day by day 25. Alternative regimens, including extended schedules, have also demonstrated clinical efficacy [28,29].

Recent meta-analyses and systematic reviews confirm that both agents significantly enhance smoking cessation outcomes compared to placebo. High-certainty evidence indicates that varenicline substantially improves 6-month quit rates (e.g., 21–25% with varenicline versus 8–10% with placebo) [13]. Varenicline also outperforms bupropion and single-form nicotine replacement (NRT) and is roughly comparable to combination NRT [13].

Cytisinicline also increases quit rates compared to placebo—for instance, 6-month abstinence was reported at 205 versus 158 per 1000 smokers (RR ≈ 1.30) [13]. A recent systematic review similarly found that cytisinicline more than doubled the odds of abstinence over a placebo [28]. In direct comparisons, no statistically significant differences in smoking cessation efficacy have been observed between cytisinicline and varenicline [13]. Thus, while varenicline is supported by a broader body of evidence and demonstrates superior outcomes compared to alternatives such as bupropion or single-form NRT, cytisinicline appears broadly comparable in efficacy.

Both medications are generally well tolerated. The most common side effect of varenicline is nausea, affecting up to 30% of users, although it is typically mild and transient [30]. Other frequent complaints include insomnia, abnormal/vivid dreams, headache, and gastrointestinal symptoms [13]. Cytisinicline’s most common side effects are gastrointestinal symptoms (e.g., nausea, vomiting, heartburn), dry mouth, and transient sleep disturbances [31]. Serious adverse events (SAEs) are rare. Cochrane reviews report similar low SAE rates with cytisinicline or varenicline versus placebo [13]. One study noted numerically fewer SAEs with cytisinicline (33 per 1000) than varenicline (49 per 1000), although confidence intervals overlapped, limiting firm conclusions.

Treatment adherence is influenced by several factors, including side effects and dosing complexity. While cytisinicline’s multiple daily doses may be burdensome, its milder side effect profile could enhance tolerability. In contrast, varenicline’s twice-daily regimen is more convenient but may be offset by higher adverse event rates.

Evidence from Oreskovic et al. [32] showed that cytisinicline had a higher adherence to the treatment plan when compared to varenicline, possibly due to the lower rate of adverse events. However, the standard 25-day cytisinicline protocol was less effective than the standard 12-week varenicline regimen in supporting long-term abstinence. Other studies have shown varenicline to be more frequently discontinued due to adverse events, yet cytisinicline has not consistently demonstrated non-inferiority to varenicline at 6-month follow-up [11].

Notably, cytisinicline appears to be much less expensive and is expected to yield more quality-adjusted life-years per dollar than varenicline in cost-effectiveness models [28].

As summarized in Table 1, both varenicline and cytisinicline are effective pharmacological options for smoking cessation. They act as partial agonists at α4β2* nicotinic acetylcholine receptors, thereby reducing withdrawal symptoms and nicotine reinforcement.

Varenicline demonstrates superior pharmacokinetic properties, higher abstinence rates, and a more robust clinical evidence base—especially in comparison to bupropion and monotherapy NRT. However, cytisinicline remains a promising and more affordable alternative. Despite its lower receptor affinity and shorter half-life, it is generally well tolerated and has shown comparable efficacy in multiple trials.

Differences in adherence, dosing complexity, and side effect profiles highlight the need for individualized treatment selection based on patient characteristics and preferences.

## 4. Comparing Varenicline and Cytisinicline According to Their Effects on Emotional Regulation, Psychological Symptoms, and Major Mental Health Concerns

### 4.1. Effects on Emotional Regulation and Psychological Symptoms

Emotional regulation plays a central role in the psychological experience of nicotine withdrawal. It refers to the processes through which individuals manage the intensity, duration, and expression of emotional responses. Impaired emotional regulation is strongly associated with psychological symptoms such as anxiety, depression, and irritability—frequent reactions during abstinence [33]. Individuals with greater difficulties in regulating negative affect tend to experience more severe withdrawal symptoms and higher relapse risk [34]. Therefore, pharmacological treatments that support emotional stabilization may not only reduce affective discomfort but also improve psychiatric outcomes and cessation success rates.

As previously discussed, both cytisinicline and varenicline alleviate withdrawal symptoms by partially stimulating α4β2* nicotinic receptors, thereby preserving dopaminergic tone and mitigating the rewarding effects of nicotine. Varenicline has demonstrated clear efficacy in this regard. In controlled trials, it significantly reduced subjective withdrawal symptoms and negative mood while enhancing positive affect and cognitive function during abstinence [35]. Meta-analyses further confirm that varenicline is not associated with increased irritability or aggression compared to placebo (OR ≈ 0.98 and 0.91, respectively) [17]. Although early safety concerns were raised regarding mood instability, large-scale randomized controlled trials (RCTs) and U.S. FDA evaluations have not found evidence of increased psychiatric risk with varenicline [17,36].

While data on cytisinicline’s neuroaffective effects are more limited, its similar pharmacodynamic profile suggests comparable outcomes. Direct comparative studies are scarce, but available trials report no meaningful differences in mood-related symptoms between cytisinicline and varenicline [37]. Thus, current evidence supports the notion that both agents can help stabilize mood during smoking cessation without increasing the risk of emotional dysregulation.

Sleep disturbances are a well-documented side effect of smoking cessation and are frequently reported with pharmacotherapy. Varenicline is consistently associated with higher rates of insomnia (OR ≈ 1.6), sleep disturbances (OR ≈ 1.63), and vivid dreams (OR ≈ 2.4) [17]. Roughly 10–15% of users report abnormal dreaming or insomnia, and among patients with psychiatric histories, sleep disruption is among the most prevalent side effects [38,39].

Cytisinicline appears to be better tolerated in this regard, with insomnia and abnormal dreams reported in fewer than 10% of participants in U.S. trials [40], likely attributable to its shorter half-life and more frequent dosing schedule.

While varenicline is highly effective, its adverse effects—particularly nausea and sleep disturbances—can negatively impact treatment adherence and emotional well-being. Cytisinicline, with its milder side effect profile, may facilitate a smoother emotional transition during cessation and improve compliance.

Both medications are effective in reducing irritability and anxiety by attenuating withdrawal; however, cytisinicline may be better tolerated by individuals with heightened emotional sensitivity.

### 4.2. Varenicline Versus Cytisinicline in Major Mental Health Concerns

Neuropsychiatric safety is a critical consideration in smoking cessation pharmacotherapy. Varenicline has been extensively evaluated in this context. Although initial reports raised concerns about its potential association with mood instability and suicidal ideation, subsequent large-scale studies, including the EAGLES trial, have found no significant increase in serious psychiatric adverse events compared to placebo or NRT [38]. In line with this, meta-analysis of trials shows varenicline does not increase suicide or attempted suicide rates, suicidal ideation, depression, or death compared to placebo independently of the presence or absence of psychiatric illness. In fact, one analysis suggests a reduced risk of anxiety (OR ≈ 0.75; 95% CI: 0.61–0.93) [17].

Preclinical studies reveal that varenicline enhances emotional processing by reducing startle responses to neutral stimuli and shows trends toward improved positive emotional categorization [41], while neuroimaging studies demonstrate reduced amygdala activity during emotional processing tasks [42].

In contrast, evidence regarding cytisinicline’s neuropsychiatric effects remains limited. A 2024 clinical study reported comparable rates of psychiatric adverse events between cytisinicline and varenicline, even among individuals with pre-existing mental health conditions [37].

Preclinical studies suggest nicotinic agents like cytisinicline have antidepressant-like effects [10,43,44], whereas its influence on anxiety remains inconclusive [45,46]. Cytisinicline’s emotional regulation benefits are supported by mechanistic studies showing its antidepressant-like properties through modulation of serotonergic pathways. Cytisinicline significantly upregulates 5-HT1A receptors, brain-derived neurotrophic factor (BDNF), and mTOR signaling in the hippocampus and amygdala—key brain regions involved in emotional regulation [44,45]. This compound reduces neuronal hyperactivity in the basolateral amygdala, a brain region consistently implicated in mood disorders [45].

Both varenicline and cytisinicline diminish the dysphoric-like state associated with nicotine withdrawal through their partial agonist activity at α4β2 nicotinic receptors, with comparable efficacy in alleviating withdrawal-induced elevations in brain reward thresholds [10]. These preliminary findings require further validation through robust clinical research. At present, neither agent appears to exacerbate depressive or anxiety disorders, although varenicline’s profile is supported by far more comprehensive evidence.

Varenicline has been cautiously used in patients with bipolar disorder (BD). In a small RCT involving euthymic individuals with BD (n = 60), varenicline significantly improved cessation outcomes (48.4% vs. 10.3% quit rate at 3 months; OR ≈ 8.1, *p* < 0.002) without triggering severe psychiatric relapses [47]. Nonetheless, vigilance is recommended, as isolated case reports describe varenicline-induced mania or hypomania [48,49,50].

In schizophrenia (SCZ) and psychotic disorders, where smoking prevalence is markedly elevated [51], varenicline has demonstrated safety and efficacy. RCTs show that varenicline significantly improves abstinence rates without worsening psychotic symptoms [52]. For example, in a trial involving stabilized patients with SCZ or BD, varenicline led to sustained abstinence at one year (45% vs. 15%) with no psychiatric deterioration. Meta-analyses similarly show that varenicline reduces cigarette consumption and expired carbon monoxide levels without increasing psychiatric adverse events [39]. A subgroup analysis of the large EAGLES trial (smokers with SCZ spectrum disorders) found that varenicline doubled abstinence odds vs. a placebo (with the number needed for treatment comparable to that for smokers without mental illness) and did not elevate neuropsychiatric events [53].

In short, varenicline is effective and appears safe in stabilized psychotic patients [52,53]. Nevertheless, some case reports have suggested a possible link between varenicline and the emergence or worsening of psychotic symptoms in individuals with pre-existing mental illness [50,54,55,56].

To date, no clinical trials have specifically assessed the efficacy or safety of cytisinicline in individuals with SCZ or BD. This absence of evidence represents a significant gap, particularly given the disproportionately high prevalence of tobacco use and nicotine dependence in these groups. While cytisinicline’s pharmacological profile suggests a lower risk of neuropsychiatric side effects compared to varenicline, such assumptions cannot substitute for empirical data.

Preclinical models suggest, however, that cytisinicline may hold therapeutic potential in SCZ and BD. For instance, cytisinicline exhibits neuroprotective effects by modulating glutamatergic signaling, particularly via down-regulation of GluN2B-containing N-methyl-D-aspartate (NMDA) receptors, which confers protection against excitotoxic injury—a mechanism relevant to schizophrenia’s pathophysiology, where NMDA receptor hypofunction is implicated [57]. Additionally, cytisinicline’s interaction with α7 nicotinic acetylcholine receptors in epileptic models shows it can reduce glutamate levels in the hippocampus and increase GLT-1 transporter expression, potentially addressing the glutamate dysregulation observed in both SCZ and BD [58].

In this context, there is a pressing need for rigorous, adequately powered randomized controlled trials to evaluate the safety, tolerability, and therapeutic potential of cytisinicline in these vulnerable populations. Addressing this research gap is essential to guide evidence-based prescribing and ensure equitable access to cessation therapies across psychiatric subgroups.

In summary, both varenicline and cytisinicline offer significant benefits in supporting emotional regulation during smoking cessation. Their shared mechanism as partial agonists at α4β2* nicotinic receptors helps mitigate irritability, anxiety, and mood lability by maintaining dopaminergic tone.

Varenicline has been more thoroughly investigated, with robust data confirming its efficacy in alleviating withdrawal-related negative affect and its relative safety in individuals with psychiatric conditions, including BD and SCZ. However, it is also more frequently associated with adverse neuropsychiatric effects such as vivid dreams, insomnia, and, in rare cases, exacerbation of underlying mental illness.

Cytisinicline, though less extensively studied, appears to offer a more favorable side effect profile—particularly regarding sleep disturbances—and may thus be better tolerated by emotionally sensitive individuals or those with existing psychiatric concerns.

Nevertheless, the lack of data in populations with severe mental illness, such as SCZ, highlights the need for further clinical trials.

Table 2 summarizes key differences in neuropsychiatric safety and emotional outcomes between the two agents, offering a useful guide for personalized treatment selection based on mental health status and tolerability.

## 5. Strengths and Limitations of the Study

This review provides an updated and clinically oriented synthesis of the evidence comparing cytisinicline and varenicline, with a particular focus on emotional regulation, psychological symptoms, and mental health vulnerabilities—an area that remains underexplored in standard smoking cessation reviews. One strength of this work is its emphasis on affective and psychiatric dimensions, which are highly relevant for real-world clinical decision-making, especially among populations with comorbid mental health conditions. In addition, this review integrates findings from recent clinical trials and meta-analyses, offering a timely perspective on two pharmacotherapies of growing global interest.

However, several limitations must be acknowledged. First, this is a narrative rather than a systematic review; therefore, while efforts were made to include high-quality and relevant studies, the selection process was not conducted following formal PRISMA guidelines or a predefined protocol. This may introduce selection bias or omit relevant studies. Second, the evidence on cytisinicline remains relatively limited, particularly in psychiatric populations, which constrains the strength of conclusions in this subgroup. Third, heterogeneity in study populations, outcome measures, and follow-up durations across the included studies may limit direct comparability. Lastly, emotional regulation is a complex construct that is not consistently operationalized across trials, making it difficult to draw definitive inferences from available data.

Future reviews using systematic methodologies and standardized outcome definitions will be necessary to confirm and extend the observations presented here.

## 6. Conclusions

This review compared varenicline and cytisinicline not only in terms of efficacy but also regarding their impact on emotional regulation, psychological symptoms, and mental health considerations. Varenicline remains the most effective first-line treatment for smoking cessation, particularly in individuals with high nicotine dependence or previous failed attempts. Cytisinicline, while less extensively studied, offers a favorable safety profile, better tolerability, and potential advantages in terms of adherence and accessibility.

Although varenicline may be preferable when long-term efficacy is the priority, cytisinicline represents a valuable alternative in contexts where cost or side effect sensitivity is considered, or when short-term pharmacotherapy is desired. Current evidence suggests that both agents support emotional stabilization during cessation. However, further research is needed, particularly regarding cytisinicline’s use in individuals with severe psychiatric comorbidities.

Clinical decisions should be individualized, considering patient preferences, psychiatric history, tolerability, and health system constraints. Pharmacotherapies for smoking cessation should ideally address not only physical dependence but also the emotional and psychological factors that sustain tobacco use. A summary of clinical applications by patient group is provided in Table 3, which may aid treatment selection based on individual needs and healthcare context.

## Figures and Tables

**Table 1:** Pharmacological overview and general comparison of varenicline and cytisinicline.

Characteristic	Varenicline	Cytisinicline
Mechanism of Action	Partial agonist at α4β2* nicotinic AChRs (high affinity); weak 5-HT_3_ binding. ^1^	Partial agonist at α4β2* nicotinic AChRs (lower affinity); nicotine analog. ^1^
Dopamine Effect	Induces ~40–60% of nicotine’s dopamine release. ^2^	Increases dopamine, likely to a lesser extent.
Pharmacokinetics	Tmax ≈ 3–4 h; t½ ≈ 24 h; minimal metabolism; 92% excreted unchanged in urine. ^3^	Tmax ≈ 1 h; t½ ≈ 4.8 h; ~64% excreted unchanged; ~42% bioavailability. ^3^
Dosing Regimen	12–24 weeks; titrated to 1 mg twice daily starting on day 8. ^4^	25-day tapering regimen (6 to 2 tablets/day); other regimens also effective. ^4^
Formulation and Route	Oral tablet or pill.	Oral tablet or pill.
Efficacy (vs. Placebo)	6-month quit rates ≈ 21–25%; superior to bupropion and monotherapy NRT; similar to combo NRT. ^5^	RR ≈ 1.3 vs. placebo; roughly doubles abstinence rates; comparable to varenicline. ^5^
Common Adverse Effects	Nausea (~30%), insomnia, vivid dreams, headache, GI symptoms. ^6^	Nausea, heartburn, dry mouth, mild sleep disturbances. ^6^
Serious Adverse Events	Rare; similar to placebo. ^7^	Rare; numerically fewer than varenicline. ^7^
Adherence Factors	Simple twice-daily dosing; adherence may be reduced by side effects. ^8^	Better tolerability; frequent dosing may reduce adherence in some. ^8^
Cost and Accessibility	Higher cost; brand-name drug.	Lower cost; higher cost-effectiveness (more QALYs per dollar). ^9^

^1^ Both act on α4β2* nicotinic acetylcholine receptors, key targets in nicotine addiction. ^2^ Dopamine release in the mesolimbic pathway is central to nicotine reinforcement. ^3^ t½ = elimination half-life; Tmax = time to maximum plasma concentration. ^4^ Standard clinical regimens: alternative dosing protocols may vary. ^5^ Based on systematic reviews and randomized controlled trials. ^6^ Frequency and severity of side effects can vary individually. ^7^ SAEs are rare for both drugs and generally comparable to placebo. ^8^ Adherence influenced by dosing complexity and tolerability. ^9^ Cytisinicline is generally more affordable and cost-effective [28].

**Table 2:** Pharmacological overview and general comparison of varenicline and cytisinicline.

	Varenicline	Cytisinicline
Withdrawal Symptom Relief	Well-documented reduction in irritability, anxiety, depressed mood, and cravings. ^1^	Expected to provide similar relief; clinical data are limited. ^1^
Impact on Emotional Stability	Improves mood and affect during abstinence; no increased aggression or irritability in large RCTs. ^2^	No evidence of emotional dysregulation; one large trial shows outcomes comparable to varenicline. ^2^
Insomnia and Sleep Disturbances	Common (~10–15%); insomnia (OR ≈ 1.6) and vivid dreams (OR ≈ 2.4). ^3^	Less frequent; reported in fewer than 10% of users. ^3^
Tolerability	Higher rates of side effects (nausea, sleep issues) may cause discontinuation.	Generally better tolerated; higher completion rates reported.
Neuropsychiatric Safety (General)	Well studied; no increased risk of serious psychiatric events in the general population (e.g., EAGLES).^4^	Limited data; no signal of increased psychiatric adverse effects so far. ^4^
Anxiety and Depression	May reduce anxiety during cessation (OR ≈ 0.75); no evidence of causing depression or suicidal ideation. ^5^	Preclinical data suggest antidepressant effects; clinical validation is lacking.
Use in Bipolar Disorder	Some case reports of mania; one RCT in euthymic patients showed efficacy without exacerbations; caution advised. ^6^	No clinical data available.
Use in Schizophrenia/Psychosis	Effective and well tolerated in stabilized patients; does not worsen psychosis; supported by RCTs and meta-analyses. ^7^	No trials conducted; no known reports of psychotic exacerbation, but data are lacking.
Overall Psychiatric Safety	Robust evidence of safety in both general and psychiatric populations.	Preliminary evidence suggests good tolerability; clinical data remain sparse.

^1^ Based on clinical trials showing symptom relief during nicotine withdrawal. ^2^ Large randomized controlled trials (RCTs) show no increase in irritability or aggression. ^3^ OR = odds ratio; indicates relative risk compared to placebo. ^4^ The EAGLES trial is a major study assessing neuropsychiatric safety of smoking cessation drugs. ^5^ Evidence supports reduced anxiety; no increased risk of depression or suicidality. ^6^ Limited bipolar disorder data; caution advised due to rare case reports. ^7^ Supported by multiple RCTs and meta-analyses in stabilized schizophrenia patients.

**Table 3:** Preferred use of varenicline or cytisinicline depending on the patient profile and clinical context.

Patient Profile	Preferred Treatment	Rationale
High nicotine dependence	Varenicline	Possible greater efficacy; longer half-life and higher receptor affinity
History of failed quit attempts	Varenicline	Superior success rates vs. placebo, bupropion, and monotherapy NRT
Anxiety, depression, irritability	Either (with caution)	Both reduce withdrawal-related affect; varenicline has RCT support; cytisinicline has fewer side effects but limited data
Bipolar disorder (euthymic)	Varenicline (with caution)	Small RCT shows efficacy; no severe exacerbations; monitor for mood symptoms
Schizophrenia or psychosis (stabilized)	Varenicline	Proven safety and efficacy in RCTs and meta-analyses; no worsening of symptoms
Sensitive to side effects	Cytisinicline	Better tolerability; lower rates of nausea, insomnia, and vivid dreams
Concern about sleep disturbances	Cytisinicline	Fewer reports of sleep-related adverse effects
Preference for short-term treatment	Cytisinicline	25-day regimen; short duration may improve adherence
Low-income or limited healthcare access	Cytisinicline	Significantly cheaper; more accessible in resource-limited settings

## Abbreviations

α4β2 nAChRs	Alpha-4 beta-2 neuronal nicotinic acetylcholine receptors
BD	Bipolar Disorder
BDNF	Brain-Derived Neurotrophic factor
C_max	Maximum Plasma Concentration
FDA	Food and Drug Administration
NMDA	N-methyl-D-aspartate receptors
NRT	Nicotine Replacement Therapy
OR	Odds Ratio
RCT	Randomized Controlled Trial
SAEs	Serious Adverse Events
SCZ	Schizophrenia
